# Rationale and design of a prospective, multicenter, phase II clinical trial of safety and efficacy evaluation of long course neoadjuvant chemoradiotherapy plus tislelizumab followed by total mesorectal excision for locally advanced rectal cancer (NCRT-PD1-LARC trial)

**DOI:** 10.1186/s12885-022-09554-9

**Published:** 2022-04-27

**Authors:** Zhengyang Yang, Xiao Zhang, Jie Zhang, Jiale Gao, Zhigang Bai, Wei Deng, Guangyong Chen, Yongbo An, Yishan Liu, Qi Wei, Jiagang Han, Ang Li, Gang Liu, Yi Sun, Dalu Kong, Hongwei Yao, Zhongtao Zhang

**Affiliations:** 1 Department of General Surgery, Beijing Friendship Hospital, Capital Medical University & National Clinical Research Center for Digestive Diseases, Beijing, China; 2 Department of Radiology, Beijing Friendship Hospital, Capital Medical University, Beijing, China; 3 Department of Pathology, Beijing Friendship Hospital, Capital Medical University, Beijing, China; 4 Department of General Surgery, Beijing Chaoyang Hospital, Capital Medical University, Beijing, China; 5 Department of General Surgery, Beijing Xuanwu Hospital, Capital Medical University, Beijing, China; 6 Department of General Surgery, Tianjin Medical University General Hospital, Tianjin, China; 7 Department of Anorectal, Tianjin People’s Hospital, Tianjin, China; 8 Department of Colorectal Cancer, Key Laboratory of Cancer Prevention and Therapy of Tianjin, Tianjin’s Clinical Research Center for Cancer, National Clinical Research Center for Cancer, Tianjin Medical University Cancer Institute and Hospital, Tianjin, China

## Abstract

**Background:**

Long course radiotherapy plus neoadjuvant chemotherapy followed by resection (total mesorectal excision, TME) has accepted widespread recognized in the treatment of locally advanced rectal cancer (LARC). Tislelizumab, an anti-PD1 humanized IgG4 monoclonal antibody, has been demonstrated with clinical activity and is approved for treating recurrent/refractory classical Hodgkin lymphoma and locally advanced/metastatic urothelial carcinoma in China. However, the safety and efficacy of long course (neoadjuvant chemoradiotherapy, NCRT) plus tislelizumab followed by TME for LARC is still uncertain.

**Methods:**

This NCRT-PD1-LARC trial will be a prospective, multicenter and phase II clinical trial designed to evaluate the safety and efficacy of LARC patients treated with long course NCRT plus tislelizumab followed by TME. This trial will consecutively enroll 50 stage II/III LARC patients (cT3N0M0 and cT1-3N1-2M0) with the tumor distal location ≤ 7 cm from anal verge at 7 centers in China. The enrolled patients will receive long course radiotherapy (50 Gy/25 f, 2 Gy/f, 5 days/week) and three 21-day cycles capecitabine (1000 mg/m2, bid, po, day1-14) plus three 21-day cycles tislelizumab (200 mg, iv.gtt, day8), followed by TME 6–8 weeks after the end of radiotherapy. The primary efficacy endpoint will be the pathological complete response (pCR) rate, which is defined as absence of viable tumor cells in the primary tumor and lymph nodes.

**Discussion:**

To our knowledge, this trial is the first multicenter clinical trial in China to assess the safety and efficacy of NCRT plus anti-PD1 therapy followed by TME to treat patients with LARC. NCRT followed by TME was recognized as the most recommended treatment against LARC while could not be completely satisfied in clinic. This study expects to provide a solid basis and encouraging outcomes for this promising combination of radiotherapy, chemotherapy and immunotherapy in LARC.

**Trial registration:**

Name of the registry: ClinicalTrials.gov. Trial registration number: NCT04911517. Date of registration: 23 May 2021. URL of trial registry record: https://www.clinicaltrials.gov/ct2/show/NCT04911517?id=BFH-NCRTPD&draw=2&rank=1.

## Background

Rectal cancer is one of the most common digestive malignant tumors in China [1, 2]. There are 253,000 new cases in China every year, accounting for 18.6% of the world [3]. With the improvement of lifestyle and living standard, the incidence rate of rectal cancer is still increasing by [4, 5]. Because of the insidious incidence and inapparent early clinical symptoms of rectal cancer, about 75% of the clinically diagnosed patients in China have been in the middle and late stage, some of them even lose the opportunity of surgery [6, 7]. Among them, low rectal cancer (the tumor distal location ≤ 7 cm from anal verge) accounted for most cases with poor quality of life because of the closer distance from the anus and lower anus preservation rate [8, 9].

At present, the standard treatment for the locally advanced rectal cancer (LARC) consisting of neoadjuvant chemoradiotherapy (NCRT) followed by total mesorectal excision (TME). Such therapeutic schedule not only can improve compliance to treatment and reduce toxicity, but also increase the rate of anus preservation and R0 resection to approach lower local recurrence rate [10, 11]. Therefore, the National Comprehensive Cancer Network (NCCN) guidelines preferred NCRT followed by TME for the treatment of LARC [12, 13]. However, the pathological complete response (pCR) rate of NCRT treating LARC patients is about 11%—15%, which is still expected to increase [14, 15]. Additionally, the overall survival (OS) rate of NCRT is not significantly higher than that of postoperative adjuvant chemotherapy according to some clinical trials [16, 17]. Consequently, novel treatment to improve the long-term survival rate of LARC is in the immediate needs.

The programmed cell death 1 (PD1) mediates immunosuppression by acting as a key immune-checkpoint receptor, while programmed cell death ligand 1 (PDL1) can engage PD1 resulting in anergy and apoptosis of T cells [18, 19] Inhibition of the interaction between PD1 and PDL1 can enhance T-cell responses further mediate antitumor activity. Based on DNA mutation pattern, colorectal cancer (CRC) consists of deficient mismatch repair (dMMR) and proficient mismatch repair (pMMR) [20, 21]. According to some clinical trials, PD-1/PD-L1 inhibitors could obtain good therapeutic effect in dMMR CRC patients and has been approved for treating dMMR CRC in clinic by the Food and Drug Administration (FDA) [22–24]. However, limited to lower proportion of dMMR (about 15%), the effectiveness of PD-1/PD-L1 inhibitors treating CRC especially LARC is still uncertain. Interestingly, radiotherapy was found to enlarge the anti-PD1/PDL1 treatment effect by promoting different links in the immune response such as activation and recruitment of T cells, promotion of dendritic cells maturation, antigen exposure and upregulation of major histocompatibility complex molecules [25, 26]. In addition, radiotherapy can also reduce tumor burden and reinvigorate exhausted T cells to strengthen the anti-PD1/PDL1 therapeutic efficacy [27, 28]. Therefore, the combination of anti-PD1 with NCRT might lead to improvement in local remission and survival in LARC.

Taking the consideration above, such NCRT-PD1-LARC phase II trial combines NCRT with tislelizumab, an anti-PD1 humanized IgG4 monoclonal antibody, to assess the safety and efficacy of this therapeutic plan for LARC patients.

## Methods

### Study design

This NCRT-PD1-LARC trial (Clinical trial number: NCT04911517) will be a prospective, multicenter, single-arm phase II clinical trial to evaluate the safety and efficacy of LARC patients treated with long course neoadjuvant chemoradiotherapy plus tislelizumab followed by TME.

**Fig. 1:**
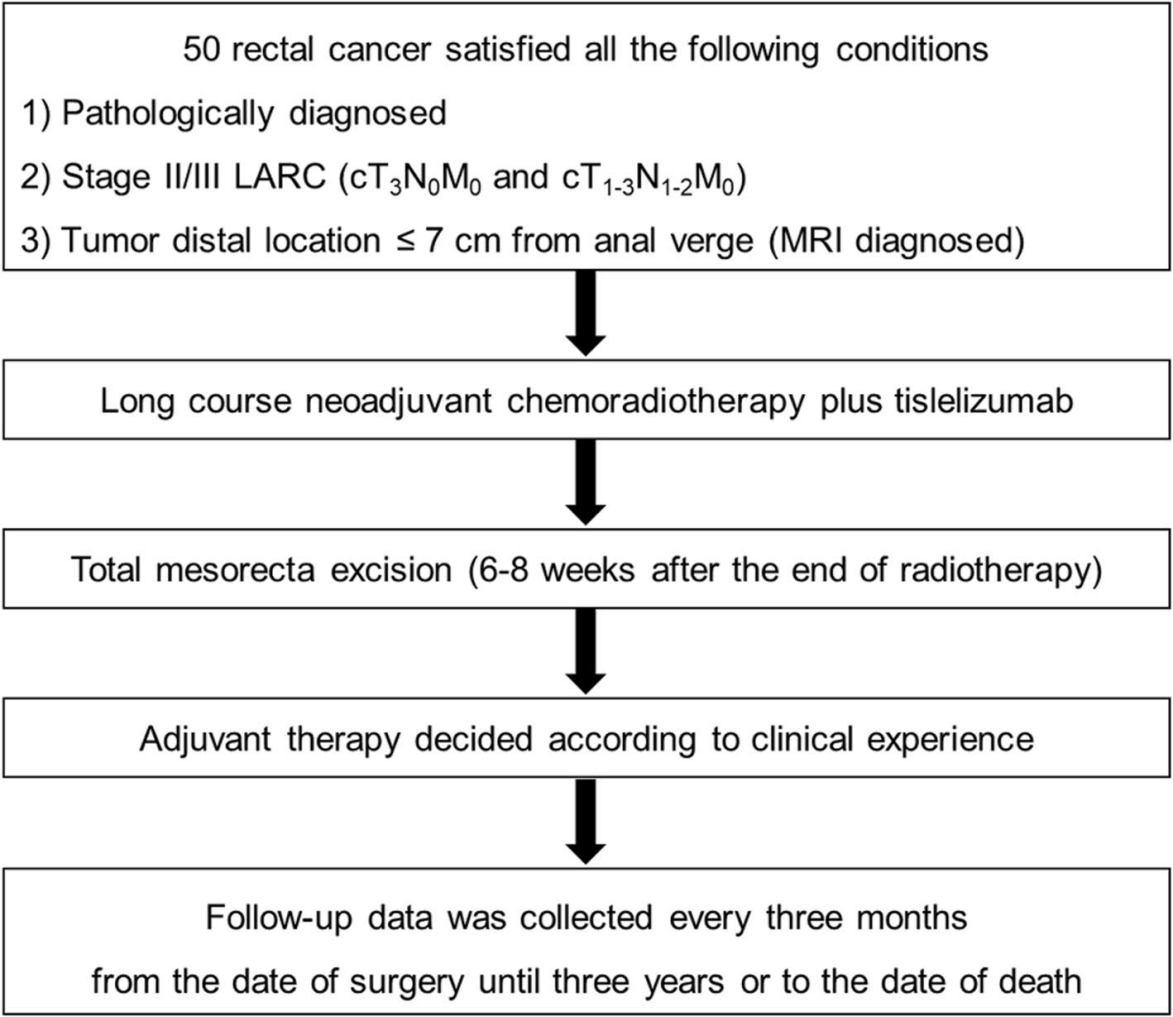
Study flow chart. (LARC, locally advanced rectal cancer; MRI, magnetic resonance imaging.)

This trial will enroll 50 patients with pathologically diagnosed LARC at 7 centers in China. This study will be in progress abide by the principles of the Declaration of Helsinki. Ethics committee of Beijing Friendship Hospital, Capital Medical University has approved the study protocol on March 30, 2021 (Approval number: 2021-P2-110–01). All enrolled patients or their designated agents will sign the informed consent. Moreover, all enrolled patients will understand the significance, methods of the study and the obligation to cooperate with the follow-up. All clinical data for each patient will be collected during visits. The flow chart of this trial is illustrated in Fig. 1.

### Patient population

Consecutive patients aged ≥ 18 years who diagnosed as LARC (cT_3_N_0_M_0_ and cT_1-3_N_1-2_M_0_) with the tumor distal location ≤ 7 cm from anal verge (determined by MRI scan) will be eligible for inclusion in this study. The major exclusion criteria are congenital or acquired immune deficiency and present or previous active malignancies (except the diagnosis of rectal cancer this time). Detailed inclusion and exclusion criteria are shown in Table 1.

**Table 1:** Inclusion and exclusion criteria

**Inclusion criteria:**
1. Patients have been fully aware of the content of this study and signed the informed consent voluntarily;
2. Patients with rectal cancers must satisfied all the following conditions:
1) Stage II/III LARC (cT_1-3_N_1-2_M_0_);
2) Tumor distal location ≤ 7 cm from anal verge (MRI diagnosed);
3. Patients regardless of gender with aged ≥ 18 years and ECOG score of 0 or 1;
4. Physical and viscera function of patients can withstand major abdominal surgery;
5. Patients are willing and able to follow the study protocol during the study;
6. Patients give consent to the use of blood and pathological specimens for study;
7. Within 28 days prior to enrolment, we must confirm a negative serological pregnancy test for child-bearing age women and they agree to use effective contraception for the duration of drug use and for 60 days after the last dose
**Exclusion criteria:**
1. Patients have a present or previous active malignancy except the diagnosis of rectal cancer this time;
2. Patients underwent major surgery within 4 weeks prior to study treatment;
3. Patients have any condition affects the absorption of capecitabine through gastrointestinal tract;
4. Patients have severe uncontrolled recurrent infections, or other severe uncontrolled concomitant diseases;
5. Patients who are allergic to any of the ingredients under study;
6. Patients with severe concomitant diseases with estimated survival ≤ 5 years;
7. Patients with present or previous moderate or severe liver and kidney damage presently or previously;
8. Patients have received other study medications or any immunotherapy currently or in the past;
9. Patients preparing for or previously received organ or bone marrow transplant;
10. Patients who received immunosuppressive or systemic hormone therapy for immunosuppressive purposes within 1 month prior to the initiation of study therapy;
11. Patients with congenital or acquired immune deficiency (such as HIV infection);
12. If patients with a history of uncontrolled epilepsy, central nervous system disease or mental disorder, the investigator will determine whether the clinical severity prevents the signing of informed consent or affects the patient's oral medication compliance;
13. Patients with other factors that may affect the study results or cause the study to be terminated midway, such as alcoholism, drug abuse, other serious diseases (including mental illness) requiring combined treatment and severe laboratory examination abnormalities
14. Pregnant or lactating women

LARC: locally advanced rectal cancer; MRI: magnetic resonance imaging; ECOG: Eastern Cooperative Oncology Group; HIV: human immunodeficiency virus

### Therapeutic schedule

**Fig. 2:**
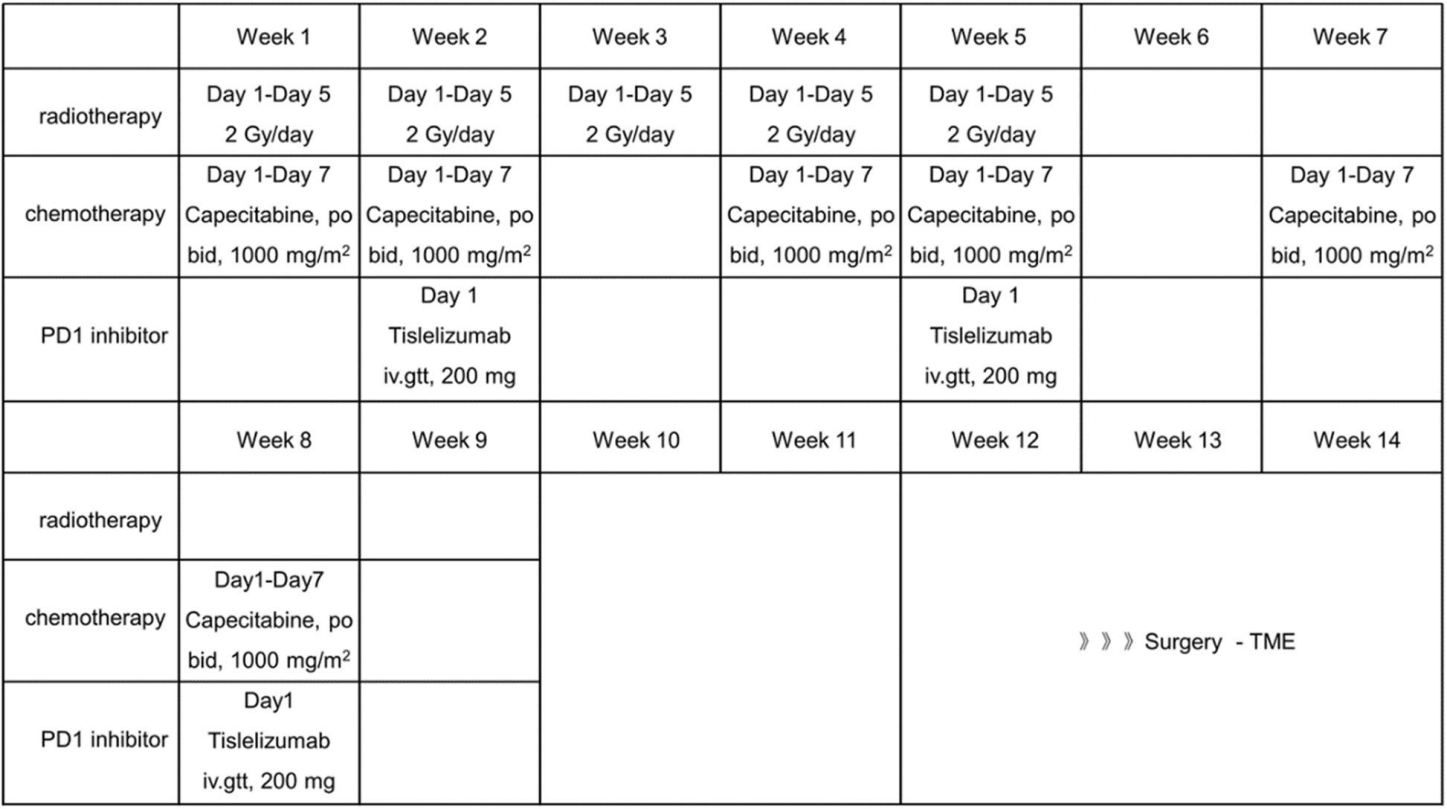
Therapeutic timelines and schedule. (PD1, programmed cell death 1; TME, total mesorectal excision.)

The enrolled patients will receive long course radiotherapy (50 Gy/25 f, 2 Gy/f, 5 days/week) in the first five weeks. As for chemotherapy and anti-PD1 therapy, the enrolled patients will be treated with three 21-day cycles capecitabine (1000 mg/m^2^, bid, po, day1-14) plus three 21-day cycles tislelizumab (200 mg, iv.gtt, day8) in the first nine weeks. Afterwards, patients will have two weeks of rest (week 10–11). While 6–8 weeks after the end of radiotherapy, patients will receive TME surgery to achieve the purpose of radical resection (week 12–14). Moreover, whether and how to use postoperative adjuvant chemotherapy will be determined according to clinical experience. The detailed therapeutic schedule and timeline is shown in Fig. 2.

### Outcomes

The primary outcome of this NCRT-PD1-LARC trial is pCR rate. All the enrolled patients will receive TME surgery 6–8 weeks after the end of radiotherapy. The resected rectal specimens will be evaluated by the pathologists who are experienced on the rectal cancer diagnosis according to the 1997 Dworak grading system [29]. The pathological results will be classified into 5 grades, while grade 0–3 will be considered as non-pCR and grade 4 represent pCR. Such outcome will be measured after the surgery as soon as possible.

**Fig. 3:**
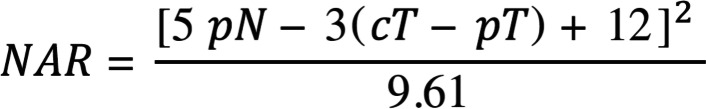
Calculation formula of the NAR score. (NAR, neoadjuvant rectal; pN, pathologic nodal stage; cT, clinical tumor stage; pT, pathologic tumor stage.)

The secondary outcome including neoadjuvant rectal (NAR) score, objective response rate (ORR), R0 resection rate, anal preservation rate, 3-year local recurrence rate (LRR), 3-year disease free survival (DFS) and 3-year overall survival (OS). The NAR score is a predictive indicator of survival after preoperative chemoradiotherapy for rectal cancer, which will be calculated according to the following formula in Fig. 3 [30]. ORR is evaluated according to Response Evaluation Criteria in Solid Tumors (RECIST) v1.1 [31]. The ORR rate is the result of complete response (CR) rate plus partial response (PR) rate. During the surgical process, surgeons will evaluate the level of cancer resection, which will be classified as R0, R1, R2 resection. The R0 resection rate will be calculated right after the surgery. Surgeons will decide whether the anal can be preserved based on the rectal cancer and intraoperative situation during the surgery. The anal preservation rate is the percentage of patients who achieve anal preservation. Additionally, the 3-year LRR, 3-year DFS and 3-year OS will be calculated after the 3-year follow-up finished.

### Adverse event

The adverse event management of this trail will strictly abide by the consensus recommendations from the Society for Immunotherapy of Cancer (SITC) toxicity management working group including dermatologic, gastroenterological, endocrine, pulmonary, rheumatologic, musculoskeletal, infusion reactions, cardiovascular, hematologic, renal, neurologic and ophthalmologic events [32]. The incidence of adverse event, management of immune-related adverse events and detailed reasons of withhold/permanently discontinue will be recorded.

### Follow up

Follow-up data was collected by professional researchers through telephone and regular outpatient visits. The follow-up period was calculated every 3 months in the first postoperative year and every 6 months after the first postoperative year from the date of surgery until three years or to the date of death.

### Quality control

In order to ensure the accuracy of diagnosis of enrolled patients, all the MR images will be uploaded to the network data registration and read by two professional radiologists. Then they will issue the standardized report (Table 2).

**Table 2:**
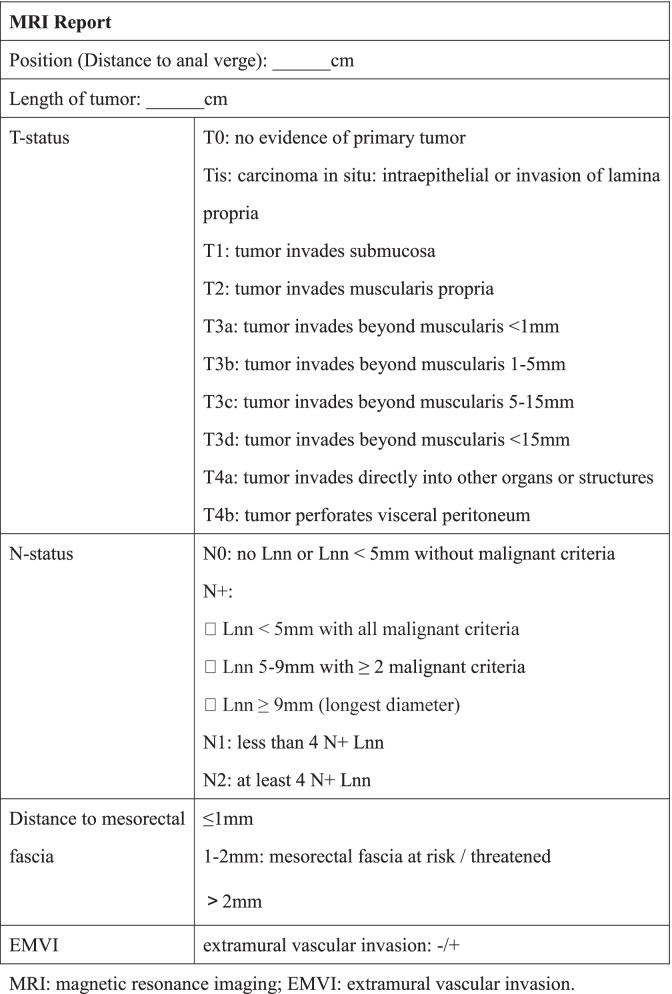
Standardized MRI report

*MRI* magnetic resonance imaging, *EMVI *extramural vascular invasion

The operation is performed by experienced surgeons who should have at least 30 cases of TME experience. All the participating surgeons needs to submit unedited operative videos of at least 3 consecutive cases. Two independent reviewers will assess the surgical appearance. Additionally, the key steps of all operations should have operation records to query.

The fresh, unopened specimen will be sent to the pathologist and processed professionally after the operation. The site of the tumor will be sliced as thinly as possible (3-5 mm slices) including up to 2 cm above and below the tumor. We will collect all these slices of enrolled patients and submit them to a specific pathologist (Prof Guangyong Chen, one of the authors.) for a final report (Table 3). The standardized pathological report is made by us with reference to Standardized Pathology Report for Colorectal Cancer, 2nd Edition [33].

**Table 3:**
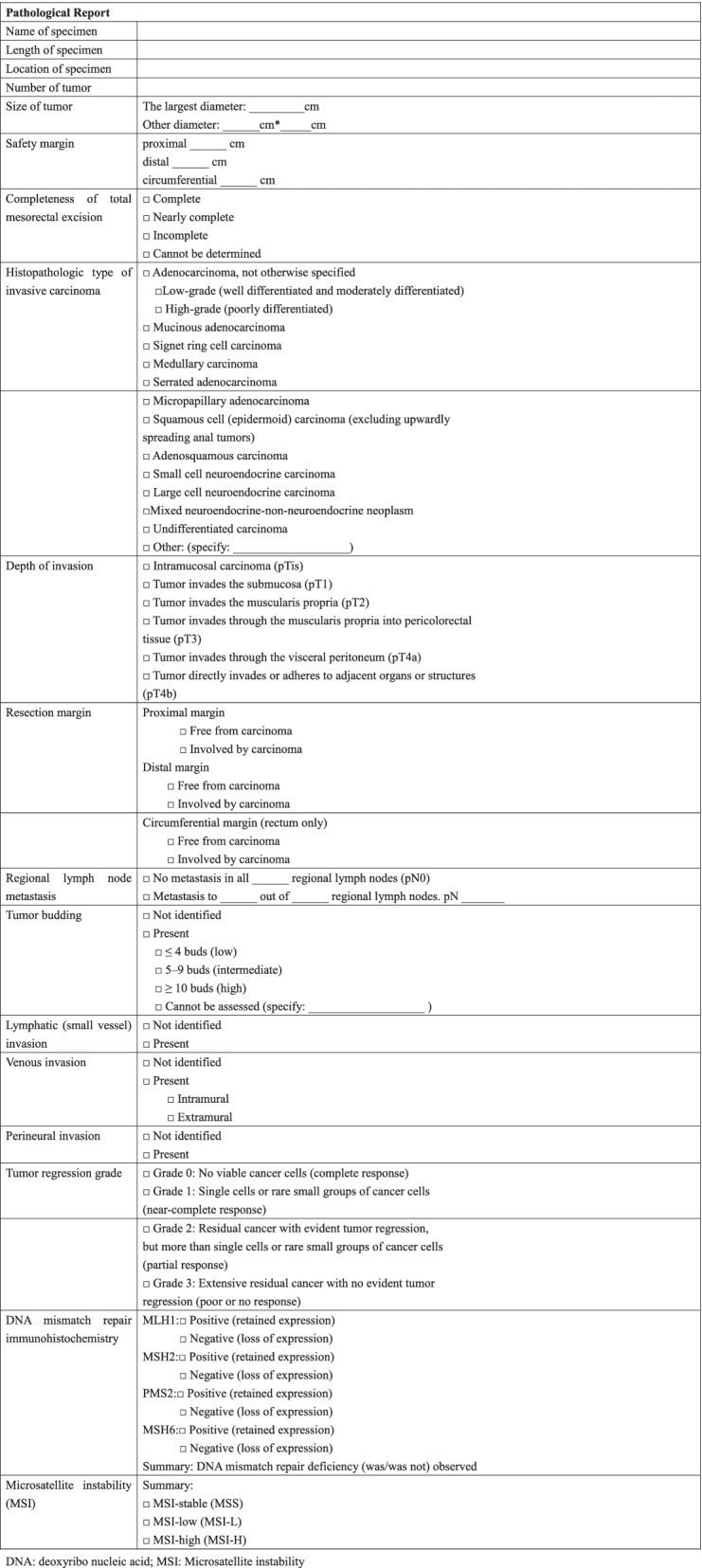
Standardized pathological report

*DNA* deoxyribo nucleic acid, *MSI* Microsatellite instability

The enrolled patients will be examined, treated and evaluated strictly according to this study protocol, while the obtained data will be recorded in the case report form. All forms must be completed, signed and dated by the person who filled in. The research supervisor will check the network database once a month to urge every center to complete the network data registration. If there is any missing data, the reason must be indicated. The follow-up will be completed by experienced professional follow-up personnel, while the collection of clinical data will be completed by gastrointestinal surgeons.

### Sample size and statistical analysis

This study is designed as a multicenter, single-arm phase II clinical trial. The pCR rate of single preoperative NCRT is assumed to be about 15% according to previous studies [14, 15]. On the other hand, the expectant pCR rate in this trail will be 40%. The required sample size was calculated to be 50 patients with 80% power and 95% confidence intervals. Moreover, 10% loss of follow-up rate is also considered. Such sample size was calculated using PASS software (version 15).

Statistical analyses will be in progress using the SPSS software (version 22.0). All analyses were 2-tailed. The confidence interval was 5–95%. p-values < 0.05 were considered as statistically significant. Continuous variables will be presented as means ± standard deviation and analyzed using an unpaired t-test with Welch’s correction. Categorical variables will be presented as number and percentage and analyzed using chi-square test with Fisher’s exact test. The Kaplan–Meier method and log-rank test were used to calculate the DFS and OS.

### Patient and public involvement

Patients and/or the public were not involved in the design, conduct, reporting or dissemination plans of this research.

## Discussion

It is widely known that the incidence rate of LARC is increasing by in recent years. Limited to closer distance from the anus and lower anus preservation rate, this kind of patients have poor postoperative quality of life. NCRT followed by TME, the current recognized treatment recommended by NCCN, may achieve limited curative effect which cannot be completely satisfied in clinic. Therefore, this study expects to provide a solid basis and encouraging outcomes for this promising combination of radiotherapy, chemotherapy and immunotherapy in LARC.

PD-1/PD-L1 inhibitors can enhance T cell activation and prevent T cells from dysfunction and apoptosis, has become more efficient in the treatment of cancer [34]. Increasing evidence suggests that dMMR patients perform significant response to anti- PD-1/PD-L1 therapy in rectal cancer [35]. Encouragingly, PD-1/PD-L1 inhibitors has been approved for treating dMMR CRC in clinic by the FDA [22–24]. Nevertheless, the proportion of dMMR in LARC is about 15% and the effectiveness of PD-1/PD-L1 inhibitors in pMMR LARC is still indeterminate. Tislelizumab is an anti-PD1 monoclonal antibody which was specifically engineered to minimize Fcγ receptor binding on macrophages, for achieving abrogating antibody-dependent phagocytosis which is identified as a reason of T-cell clearance and further potential resistance to anti-PD1 therapy [36, 37]. Furthermore, tislelizumab has been approved for treating recurrent/refractory classical Hodgkin lymphoma and locally advanced/metastatic urothelial carcinoma in China. This study concentrates on the safety and efficacy of conventional therapy plus PD-1/PD-L1 inhibitors in all types of LARC patients, not limited in dMMR.

Increasing studies prove the superiority of NCRT in comparison to postoperative adjuvant chemotherapy, which adjust maximized treatment before surgery to acquire better compliance, fewer toxicity profiles, and superior pCR rates. Thereinto, long course radiotherapy (50 Gy/25 f, 2 Gy/f, 5 days/week) combine chemotherapy (capecitabine) is a more frequently chosen NCRT plan with fewer adverse events [38, 39]. Considering the combination of PD-1 inhibitors, such safer NCRT therapeutic plan might be appropriate. Ingeniously, it has been widely confirmed that radiotherapy combined with PD1/PDL1 inhibitors has synergistic antitumor effect. A phase I clinical trial (KEYNOTE-001) indicated that patients with advanced non-small cell lung cancer who had received radiotherapy before treating with Pembrolizumab, a PD-1 inhibitor, achieved longer progression-free survival and overall survival compared with those without radiotherapy [40]. Previous studies testified that radiotherapy up-regulate the expression of PD-L1 in tumor cells and macrophages, further reversed T cells depletion and apoptosis and make T cells immune response fully to fight tumor [41]. Moreover, the combination of radiotherapy and PD1/PDL1 inhibitors could induce the immune response of tumor antigen-specific T cells (CD8^+^ T cells), which enhanced the effector function of cytotoxic T lymphocyte (CTL) in tumor microenvironment to achieve more superior antitumor effect [42]. Consequently, the injection timing of tislelizumab was started form week 2 in order to achieve synergistic antitumor effect for better curative outcomes.

Inevitably, several limitations in this study need to be discussed. First, to explore the curative effect of the treatment strategy, we design this single-arm study. Though the single-arm design might provide a little weaker level of evidences owing to the lack of randomness and another control arm like NCRT group. A number of single-arm clinical studies on neoadjuvant therapy combined with immunotherapy are being conducted in order to assess clinical outcomes more quickly [43, 44]. However, there are so many patients suffering from advanced rectal cancer, it is very urgent to find out an effective treatment. Based on the number of visits at each research center, all the patients involved in this single-arm study will be operated and we can get the primary outcome in less than a year so that we can figure out how effective this treatment is quickly. If we get a satisfied result, we will carry out a further controlled trial (RCT) with more sites to evaluate the safety and efficacy of adding anti-PD1 therapy with NCRT to treat patients with LARC more credibly. Secondly, this study focuses only on Chinese LARC patients without other ethnic groups and its sample size is a little small. Moreover, different plan of chemotherapy and radiotherapy in NRCT might become a potential bias to calculate the sample size.

## Conclusion

To our knowledge, this is the first multicenter clinical trial in China to assess the safety and efficacy of NCRT plus anti-PD1 therapy followed by TME to treat patients with LARC. Despite there are some limitations because of the design of the one-arm study, we can get the primary outcome more quickly and we hope that this study can provide us positive outcomes and further encourage phase III trials.

## Data Availability

Not applicable.

## Abbreviations

LARC: Locally advanced rectal cancer
NCRT: Neoadjuvant chemoradiotherapy
TME: Total mesorectal excision
NCCN: National Comprehensive Cancer Network
pCR: Pathological complete response
OS: Overall survival
PD1: Programmed cell death 1
PDL1: Programmed cell death ligand 1
CRC: Colorectal cancer
dMMR: Deficient mismatch repair
pMMR: Proficient mismatch repair
FDA: Food and Drug Administration
NAR: Neoadjuvant rectal
ORR: Objective response rate
LRR: Local recurrence rate
OS: Overall survival
DFS: Disease free survival
RECIST: Response Evaluation Criteria in Solid Tumors
CR: Complete response
PR: Partial response
SITC: Society for Immunotherapy of Cancer
MRI: Magnetic resonance imaging
ECOG: Eastern Cooperative Oncology Group
HIV: Human immunodeficiency virus
EMVI: Extramural vascular invasion
DNA: Deoxyribo nucleic acid
MSI: Microsatellite instability
